# Moonlighting of *Helicobacter pylori* catalase protects against complement-mediated killing by utilising the host molecule vitronectin

**DOI:** 10.1038/srep24391

**Published:** 2016-04-18

**Authors:** Corinna Richter, Oindrilla Mukherjee, David Ermert, Birendra Singh, Yu-Ching Su, Vaibhav Agarwal, Anna M. Blom, Kristian Riesbeck

**Affiliations:** 1 Clinical Microbiology, Department of Translational Medicine, Lund University, SE-205 02 Malmö, Sweden; 2 Medical Protein Chemistry, Department of Translational Medicine, Lund University, SE-205 02 Malmö, Sweden

## Abstract

*Helicobacter pylori* is an important human pathogen and a common cause of peptic ulcers and gastric cancer. Despite *H. pylori* provoking strong innate and adaptive immune responses, the bacterium is able to successfully establish long-term infections. Vitronectin (Vn), a component of both the extracellular matrix and plasma, is involved in many physiological processes, including regulation of the complement system. The aim of this study was to define a receptor in *H. pylori* that binds Vn and determine the significance of the interaction for virulence. Surprisingly, by using proteomics, we found that the hydrogen peroxide-neutralizing enzyme catalase KatA is a major Vn-binding protein. Deletion of the *katA* gene in three different strains resulted in impaired binding of Vn. Recombinant KatA was generated and shown to bind with high affinity to a region between heparin-binding domain 2 and 3 of Vn that differs from previously characterised bacterial binding sites on the molecule. In terms of function, KatA protected *H. pylori* from complement-mediated killing in a Vn-dependent manner. Taken together, the virulence factor KatA is a Vn-binding protein that moonlights on the surface of *H. pylori* to promote bacterial evasion of host innate immunity.

*Helicobacter pylori* is a spiral-shaped Gram negative bacterium that specifically colonises the human stomach^1^. It has been estimated that over half of the global population is colonised by this pathogen^2^. Even though infections can remain asymptomatic, colonisation with *H. pylori* will lead to gastritis and peptic ulcers in at least 10% of cases. Approximately 1% of infected individuals develop gastric adenocarcinoma, which represents up to 80% of all gastric cancers^3^. Infection with *H. pylori* provokes a strong innate as well as cellular and humoral adaptive response. Despite the immunogenicity of *H. pylori*, however, the host is usually unable to clear the infection. Known mechanisms by which *H. pylori* subverts the host’s immune response include avoidance of recognition by Toll-like receptors^4^, survival in macrophages^5^, and inhibition of both T cell activation and memory T cell responses^6^,^7^ to name only a few.

‘Hijacking’ of host molecules is a strategy used by several bacterial pathogens to facilitate adhesion and/or to evade the host’s immune response^8^,^9^. In this respect, one particularly interesting host factor is vitronectin (Vn), an abundant component in human plasma and the extracellular matrix (ECM). Vn is a multifunctional glycoprotein (75 kDa single-chain or 65 kDa plus 10 kDa two-chain form), which can be present in a monomeric or an active multimeric state^10^. The molecule is composed of the N-terminal somatomedin-B domain followed by an RGD motif, which is recognised by integrins. The central part is dominated by three hemopexin-like domains and a fourth hemopexin-like domain is located at the C-terminus. The heparin binding domain 3 (HBD-3), also located at the C-terminal end, is the major binding site for most studied bacterial Vn-binding proteins^8^. There are two more putative heparin-binding sites (*i.e.*, HBD-1 and HBD-2) located in the central part of the protein, but their relevance for heparin binding is equivocal. Vn is recognised by various cell surface receptors such as integrins or proteoglycans thereby functioning in cell adhesion, migration, and tissue remodelling^9^. Additionally, Vn is involved in the regulation of the complement system^9^. The complement system is composed of more than 50 soluble and membrane-bound factors and is considered a major player of innate immunity. Activation induces a proteolytic cascade, which eventually leads to the induction of the terminal pathway^11^. Here, Vn interferes with the formation of the membrane attack complex (MAC) by inhibiting the C5b-C7 complex and polymerisation of C9, subsequently preventing cell lysis^8^. Several pathogenic bacteria including *Haemophilus influenzae*, *Neisseria meningitidis*, and *Pseudomonas aeruginosa* have been shown to bind Vn, thereby increasing their complement resistance or ability to adhere to host tissues^8^.

*H. pylori* binds Vn, but the proteins involved and the importance of this interaction for virulence have not been elucidated^12^. In this study, we identified Vn-binding properties of the *H. pylori* catalase KatA and characterised the interaction on the molecular level. Furthermore, we showed that Vn binding by *H. pylori* KatA increases complement resistance and, thus, is an important factor in the evasion of the innate immune response.

## Results and Discussion

### *H. pylori* catalase KatA binds Vn

**Figure 1:**
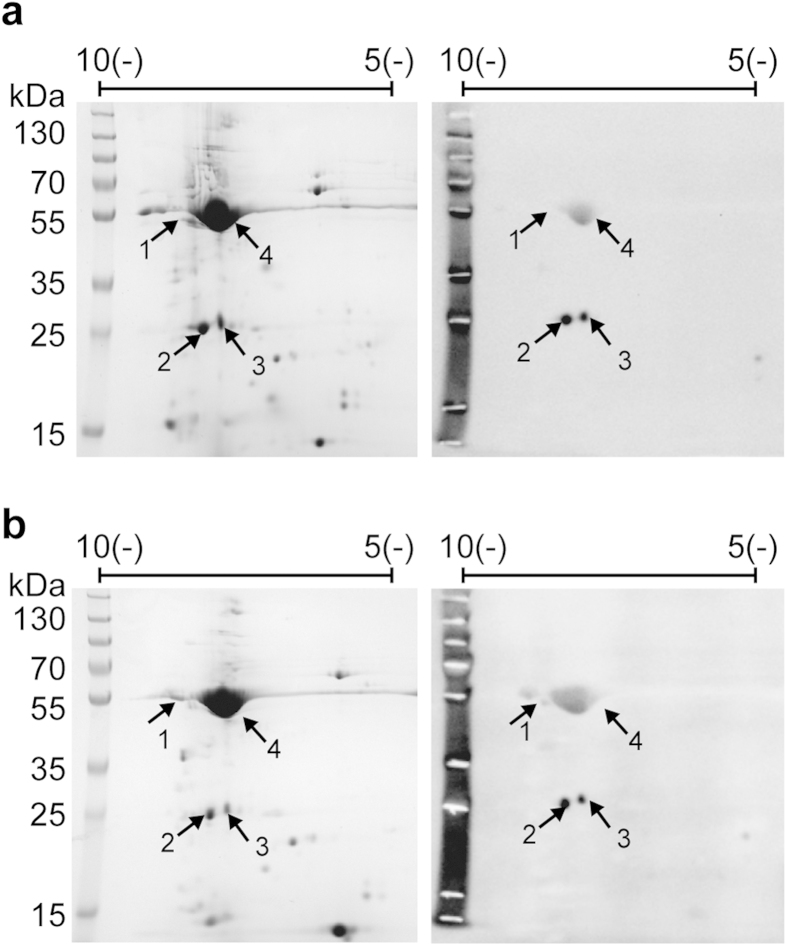
Identification of *H. pylori* Vn-binding proteins. Outer-membrane fractions of strains CCUG18943 (**a**) and KR697 (**b**) were subjected to 2D-analysis. Left panels show Coomassie stained gels, panels to the right show detection of Vn-binding proteins by far-Western blotting. Vn-binding spots were identified by MALDI-TOF as KatA (1) and UreA (2 and 3). Spot 4 corresponds to UreB.

The first aim of this study was to identify the major Vn-binding protein in *H. pylori* since the bacterium has previously been shown to bind Vn with varying binding capacity for different isolates^12^. We therefore tested binding of Vn, which was purified from sera obtained from healthy human volunteers, to 13 clinical strains from our collection (Supplementary Fig. S1 and Supplementary Table 1). For further analysis we chose three *H. pylori* with strong (CCUG18943; 92% binding), intermediate (KR697; 69%), and weak Vn binding (KR497; 15%). Outer membrane fractions of these three strains were prepared and subjected to 2D-gel electrophoresis followed by far-Western blotting against human serum Vn (Fig. 1a and b). Three Vn-binding protein spots were detected in both *H. pylori* CCUG19843 (Fig. 1a) and KR697 (Fig. 1b). A fourth intense spot corresponded to urease subunit B (UreB). No Vn-binding proteins were detected in the weak Vn-binding strain KR497 (data not shown). Interestingly, subsequent MALDI-TOF MS analysis of proteins in KR697 and CCUG18943 identified spots 1 and 2 as two isoforms of *H. pylori* urease subunit A (UreA) and spot 3 as catalase KatA. To further verify our findings, we performed a pull-down experiment, in which the outer membrane fraction of *H. pylori* CCUG18943 was subjected to a Vn-coupled Sepharose column (data not shown). MALDI-TOF MS identified KatA, but neither UreA nor UreB could be detected in this assay, despite the fact that mild conditions (pH 7.4, 140 mM NaCl) were used during the pull-down. It is possible, however, that the conditions chosen in the pull-down experiment were not optimal for urease and we therefore, at this point, cannot exclude that urease binds Vn. However, the fact that KatA could successfully be identified as a Vn-binding protein in two different experimental setups suggested a significant interaction between the two proteins and prompted us to focus our attention on the characterisation of KatA in the sequel of this study.

The catalase KatA is an important virulence determinant of *H. pylori* with designated enzymatic functions^13^,^14^, and the identification of KatA as putative Vn-binding partner was at first sight surprising. The phenomenon of additional, unrelated functions in (often highly conserved) proteins has, however, been described previously, and is now known as ‘moonlighting’^15^. Examples of moonlighting proteins are the superoxide dismutase (SOD) of *Mycobacterium avium*, which exhibits additional functions as an adhesin targeting aldolase, glyceraldehyde-3-phosphate dehydrogenase (GAPDH) and cyclophilin A on epithelial cells, thereby promoting endocytosis^16^ or the *H. influenzae* protein F, an ABC transporter, which also binds laminin and facilitates adhesion^17^.

**Figure 2:**
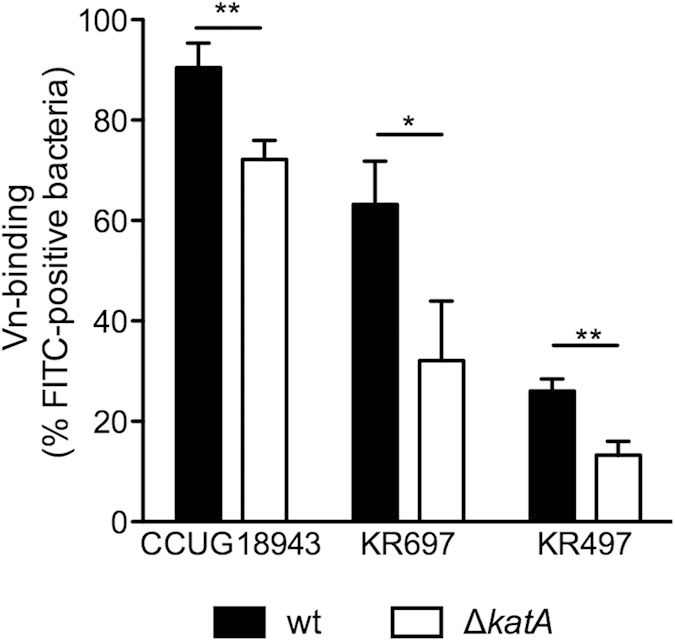
*H. pylori* D *katA* strains are impaired in Vn-binding. Wt and Δ*katA* mutants of *H. pylori* strains CCUG18943, KR697, and KR497 were incubated with 3 μg of serum Vn. The percentage of Vn-binding was measured by flow-cytometry using FITC-labeled anti-Vn Ab. Statistical significance was determined using Students *t*-Test, where (*) equals *p* ≤ 0.05 and (**) equals *p* ≤ 0.01. Data presented are the mean and SD of three independent experiments performed in technical duplicate.

*H. pylori* KatA, like other catalases, is a predicted cytoplasmic protein with no signal-sequence for any known transport pathway. However, presence of KatA in the periplasm and even surface location has been proposed^18^,^19^, even though the means of translocation remain elusive. Obviously, surface location of KatA would be essential for the interaction with Vn. Thus, we wanted to verify the KatA-dependent Vn binding with intact bacteria and constructed *katA* deletion mutants in *H. pylori* strains CCUG18943 and KR697, as well as in the weak Vn-binding KR497. All *H. pylori* Δ*katA* strains showed significantly less binding to Vn when compared to the wild type (wt) counterparts in flow cytometry (Fig. 2). Deletion of *katA* resulted in 21% reduction in binding of Vn to *H. pylori* CCUG18943, whereas a 50% reduction in Vn binding was observed with the two strains KR697Δ*katA* and KR497 Δ*katA* when compared to their respective wt (Fig. 2). Taken together, our data provided additional evidence for surface localisation of KatA and furthermore showed that KatA is relevant for Vn binding to whole bacteria.

Deletion of *katA* did not completely abolish Vn binding and the relative impact of the deletion on Vn binding varied between isolates, suggesting that *H. pylori* has additional Vn-binding proteins. The concept that one bacterial species possesses multiple proteins targeting the same ligand is well established. *N. meningitidis* outer membrane proteins Meningococcal surface fibril (Msf) and opacity protein (Opc) both bind Vn and contribute to serum resistance^20^,^21^. The presence (or absence) of other Vn-binding proteins in particular *H. pylori* strains could explain the observation that different isolates exhibit variable Vn-binding capacity. *H. pylori* lacks clonality and substantial differences between genomes exist^3^. Other possible explanations include variation in surface expression of the relevant proteins or changes in the amino acid sequence, which lead to lower affinity for Vn.

### *Helicobacter pylori* KatA binds Vn using a unique binding site

**Figure 3:**
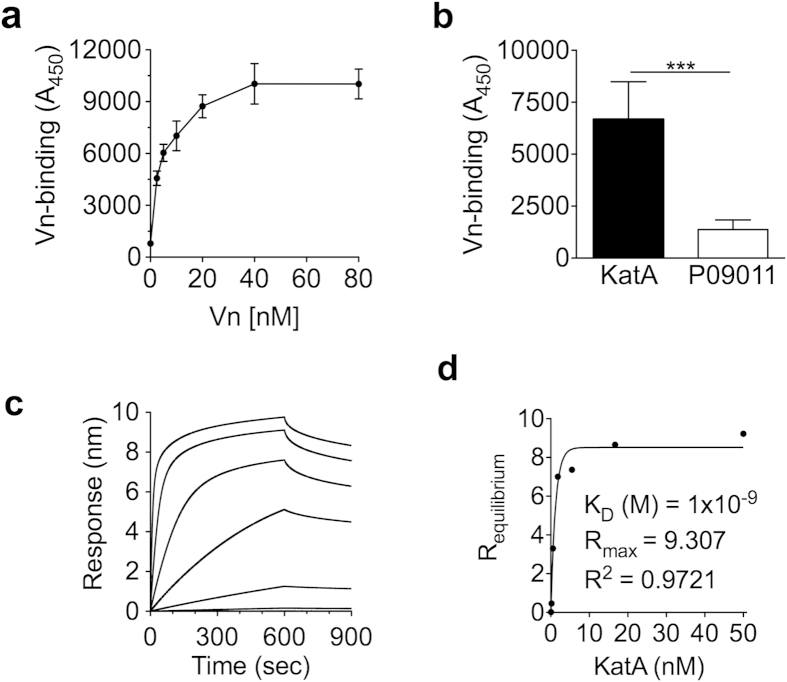
KatA is a Vn-binder with high affinity. (**a**) Binding of recombinant KatA to Vn^80–396^ was determined by ELISA. Plates coated with 100 nM KatA were incubated with increasing concentrations (2.5–80 nM) of Vn. Protein P09011 from *Haemophilus influenzae* incubated with 20 nM Vn served as negative control (inset). Statistical significance was determined using Student’s *t*-test, where (***) equals *p* ≤ 0.001. Data presented are the mean and SD of at three independent experiments performed in technical triplicates. (**b**) Binding of KatA to monomeric (native) or polymeric (activated) Vn. Plates coated with 50 nM Vn were incubated with increasing concentrations (2.5–80 nM) of KatA. (**c**) Vn^80–396^ was immobilised on amine reactive AR2G sensors and biolayer-interferometry was performed with KatA as analyte using a 1:3 dilution series (0.055–50 nM). (**d**) The affinity constant (K_D_) was calculated from steady state analysis based on R equilibrium values.

Next, we wanted to characterise the KatA-Vn interaction at the protein level. We performed an ELISA using full length recombinant KatA and a dilution series (2.5–80 nM) of Vn^80–396^, which spans most known bacterial binding sites. Binding of KatA and Vn was dose-dependent and saturable, confirming a specific interaction (Fig 3a). To exclude any effects resulting from trace amounts of *Escherichia coli* proteins, P09011 from *H. influenzae*^22^, which was purified using the same protein expression and purification protocol as for KatA, served as negative control for Vn binding (Fig. 3a, inset). Furthermore, we wanted to reveal whether KatA preferentially targets native (monomeric) Vn or the activated polymeric form. Binding of KatA to either of the two forms of Vn was investigated by ELISA and showed that KatA bound both monomeric and polymeric Vn (Fig 3b). However, binding to the activated, polymeric Vn was nearly twice as strong. Given that *H. pylori* does not typically enter the bloodstream and Vn occurs predominantly in the activated form in tissues and the ECM, targeting mainly polymeric Vn is certainly an advantage for the bacterium. A similar preference for activated Vn has been described for other bacteria including *Streptococcus pneumoniae* and *N. meningitidis*^20^,^23^. It should be noted that the activated form of Vn undergoes conformational changes and partial unfolding, and thereby exposes potential binding sites^10^, which is likely a reason why the activated form is predominantly targeted by bacteria.

To obtain more detailed information on the binding kinetics, BioLayer Interferometry was performed using Vn^80–396^ as ligand and dilutions of full length KatA as analyte (Fig. 3c,d). We obtained fast association rate constants (k_on_ [1/Ms] ≈ 1 × 10^6^) combined with moderate to slow dissociation rates (k_off_ [1/s] ≈ 3 × 10^−4^). Accordingly, steady state analysis revealed a strong affinity (K_D_ = 1 × 10^−9^ M), which is higher than the affinities determined for other Vn-binding proteins such as *Moraxella catarrhalis* ubiquitous surface protein (Usp) A2 (2.34 × 10^−8^ M)^24^ and *H. influenzae* protein F (1.28 × 10^−8^ M)^22^.

**Figure 4:**
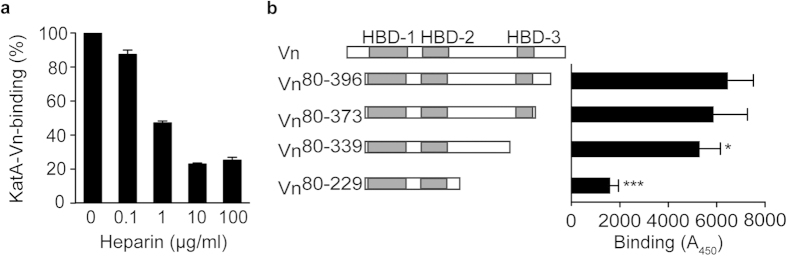
KatA interacts with Vn via an unusual binding site. (**a**) Inhibition of the KatA-Vn interaction by heparin was tested by ELISA. KatA (100 nM) was immobilised on plates and Vn^80–396^ was added after being pre-incubated with different concentrations of heparin. (**b**) The ability of Vn-fragments to bind KatA was tested by ELISA. KatA (100 nM) was immobilised and incubated with different Vn-fragments at a concentration of 20 nM. Data shown are the mean and SD of three independent experiments performed in technical triplicate. Statistically significant differences were determined using one-way ANOVA and Bonferroni’s post-test where (*) equals *p* < 0.05 and (***) equals *p* < 0.001.

There are two major bacterial binding regions within the Vn molecule. Most pathogens bind to the C-terminal HBD-3^25^. The second binding region, used by *N. meningitidis* Vn-binding proteins Msf and Opc is located at the N-terminal end of the Vn molecule and the importance of sulphated tyrosine residues Y56 and Y59 was demonstrated for Opc binding to Vn^26^,^27^. Since KatA bound to Vn^80–396^, we suspected an interaction with HBD-3 without involvement of the N-terminus. When bacterial Vn-binding proteins utilise the C-terminal HBD-3, binding can be effectively blocked by heparin. Thus, we tested the influence of heparin on the Vn-KatA interaction in an ELISA (Fig. 4a), and pre-incubated Vn with increasing concentrations of heparin (0.1–100 μg/ml). Heparin inhibited binding of KatA to Vn by only 80%, and higher heparin concentrations had no stronger inhibitory effect (data not shown). This result suggests that, in contrast to most other known Vn-binding proteins, KatA does not use HBD-3 as a major binding site but may have additional binding region(s). To narrow down the binding site, binding of KatA to a range of truncated Vn molecules was tested by ELISA (Fig. 4b). KatA was immobilised in 96 well plates and incubated with different Vn-fragments, which were detected using anti-Vn antibodies (Abs). Removal of the C-terminus distal of HBD-3 (Vn^80–373^) had no significant effect on binding when compared to Vn^80–396^. Similarly, Vn^80–339^, which lacks the whole HBD-3, showed only a minor decrease in binding KatA, which is in agreement with our observation that heparin had only a partial inhibitory effect. Strikingly, binding was substantially impaired with fragment Vn^80–229^ (Fig. 4b). Reciprocal experiments, i.e., when Vn-fragments were immobilised, incubated with KatA and binding was detected using anti-KatA Abs, gave similar results (data not shown). We observed some residual binding of Vn^80–229^ and cannot fully exclude involvement of residues located further upstream. However, the primary binding site for KatA is located within amino acids 229 and 339 of the Vn-molecule, a region not involved in interactions with any bacterial Vn-binding proteins described to date. The fact that heparin has an inhibitory effect on the interaction, even though HBD-3 is not the primary binding site, can be explained with the model structures of Vn prepared by two independent research groups^28^,^29^. Both models proposed a close proximity of the central domain and the C-terminal HBD resulting in a large inter-domain contact surface, which contains the putative heparin-binding groove. Given the spatial proximity between HBD-3 and our predicted KatA binding region within the central domain, it is feasible that bound heparin sterically disrupts the interaction between KatA and Vn rather than occupying the binding site itself. Docking of heparin to Vn^28^ supports this hypothesis. The use of an alternative binding site also explains why our kinetic data differed from data obtained for UspA2 and protein F, which both use HBD-3 as binding site^22^,^24^. In summary, *H. pylori* KatA uses a not previously characterised bacterial binding site on the Vn-molecule, which allows a high affinity interaction.

### The extended loop of KatA interacts with Vn

**Figure 5:**
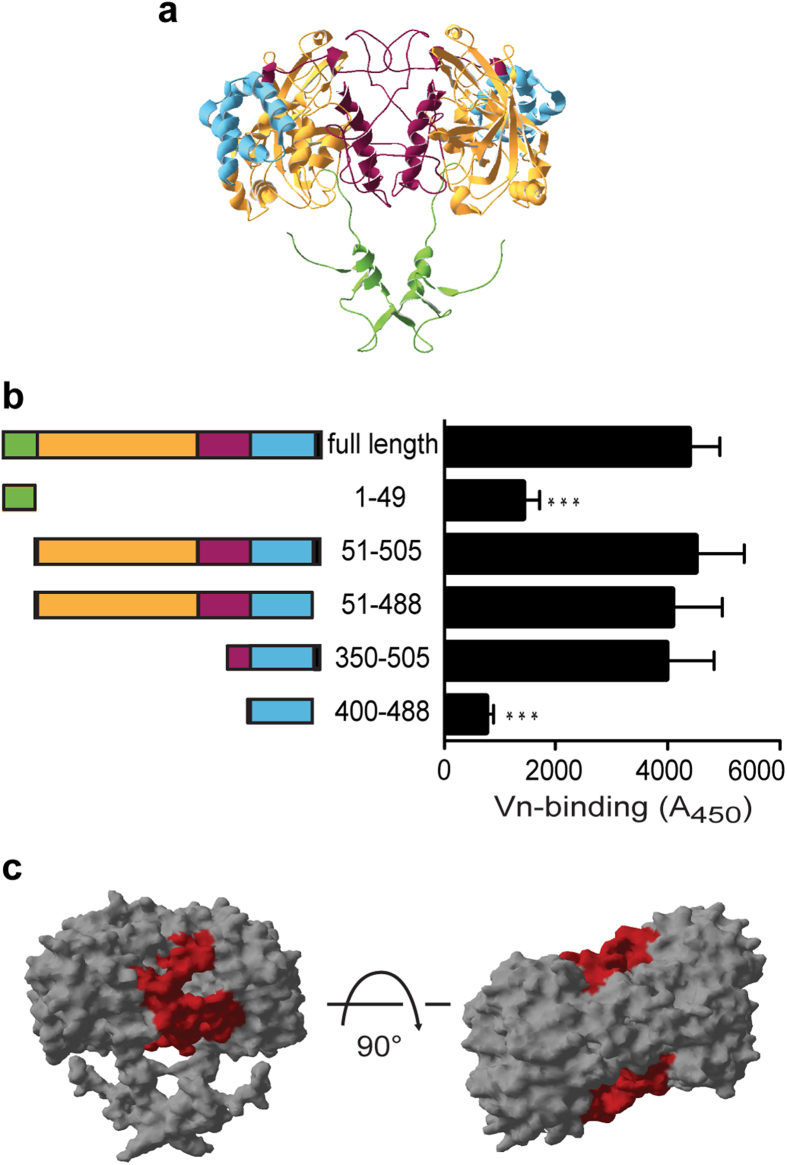
KatA binds vitronectin with its extended wrapping loop. Dimer of *H. pylori* KatA. Each monomer consists of an N-terminal arm (green), a central β-barrel domain (orange), the wrapping-loop (magenta), and the helical domain (blue). The C-terminal amino acids 492–505 are missing in the structure. (**b**) KatA-fragments were designed based on structural features shown in (**a**) and the Vn-binding capacity was compared to full length KatA using 20 nM Vn in an ELISA. Data shown are the mean and SD of at three independent experiments performed in technical triplicate. Statistically significant differences were determined using one-way ANOVA and Bonferroni’s post-test where (***) equals *p* < 0.001. (**c**) Surface structure-model of KatA dimer-surface depicting the area of the Vn-binding site (magenta).

*H. pylori* KatA, like other catalases, forms tetramers^30^. Each KatA monomer consists of 505 amino acids and exhibits the topology typical for small subunit, clade 3 catalases (Fig. 5a): an N-terminal protruding arm (aa 1–55), involved in formation of the homo-multimer, a central β-barrel domain (aa 56–315), and a C-terminal helical domain (aa 429–500), which is linked to the β-barrel domain by an extended ‘wrapping’ loop (aa 316–428)^30^. The unique feature of *H. pylori* catalase is a four lysine-motif at the C-terminus (aa 501–505), the function of which is at present unknown. Based on this topology a range of KatA fragments were designed, and their ability to interact with recombinant Vn was investigated by ELISA (Fig. 5b). KatA^1–49^ did not show any binding to Vn and, accordingly, KatA^51–505^ was not impaired in binding Vn when compared to the full-length protein (Fig. 5b). These results indicate that the N-terminal arm of KatA is dispensable for the interaction with Vn. This was expected, since the N-terminus is not exposed in the tetramer. As mentioned above, *H. pylori* catalases comprise a tetra-lysine motif as part of an unstructured C-terminus. It was proposed that this motif could be involved in the transport of KatA to the surface or in anchoring KatA to the membrane^30^; however, neither of these theories has to date been proven. We wanted to know, whether this C-terminal motif mediates the interaction with Vn and tested a fragment comprising amino acids 51–488 (Fig. 5b). Indeed, there was a slight reduction in binding to Vn, albeit not statistically significant. Therefore, the C-terminal tetra-lysine motif is, if at all, only marginally involved in Vn binding. Removal of the central β-barrel and the N-terminal part of the wrapping loop resulted in a small, but not statistically significant, reduction in binding (Fig. 5b). Finally, we tested KatA^400–488^, which additionally lacked most of the wrapping loop. When this KatA fragment was included in our analysis a strong reduction of Vn binding was observed when compared to the full length KatA. Therefore, the region, which binds Vn, most likely is located within the central part of the extended wrapping loop of KatA (Fig. 5b,c). Disordered regions of proteins, which include loops, have a high propensity for involvement in protein-protein interactions^31^, a recent example being the fibronectin protein FNE in *Streptococcus equi*^32^.

### KatA surface exposure differs between isolates and is correlated to Vn binding

**Figure 6:**
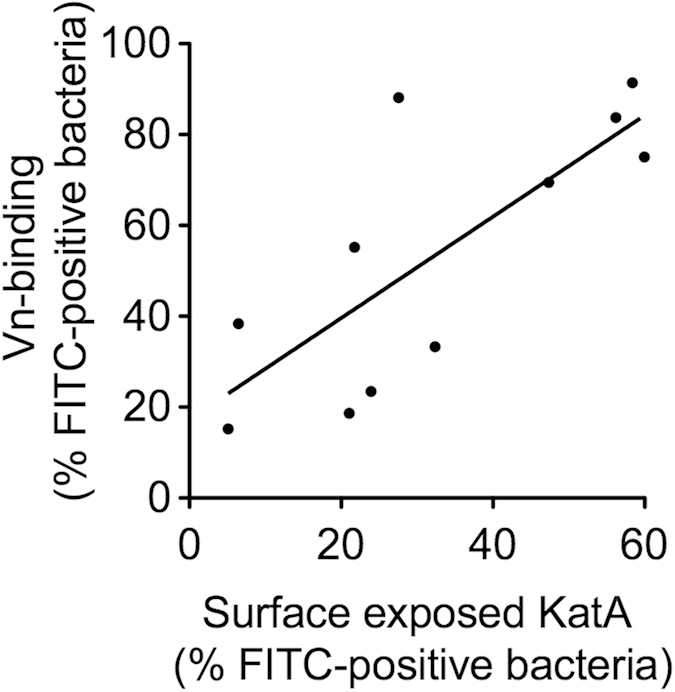
Surface exposure of KatA correlates with Vn binding capacity. Eleven *H. pylori* isolates were tested for their Vn binding capacity and KatA surface density in separate flow cytometric assays. Data shown are the mean of at least three independent experiments. Linear regression was performed for all data points.

Having identified the binding site for Vn within a flexible region, which are often less conserved, we wondered whether the differing binding properties of our three *H. pylori* isolates (Supplementary Fig. S1 and Fig. 2) could be attributed to diverging amino acid sequences. Interestingly, alignment of the KatA sequences of our three isolates and the reference strain *H. pylori* 22695 showed no differences in the relevant region between amino acid 350 and 400 (Supplementary Fig. S2). The differences in Vn binding might further be explained by a varying presence of KatA on the bacterial surface. We therefore employed flow cytometry analysis to determine KatA surface exposure in 11 of our previously tested strains including *H. pylori* CCUG18943, KR697, and KR497 (Supplementary Table 1). When the Vn-binding capacity was plotted against KatA surface expression we found a clear correlation of the two, *i.e.*, overall, high Vn binders were also highly positive for KatA and vice versa (Fig. 6). We attribute the fact that some of our *H. pylori* isolates slightly diverged from this general pattern to the presence of other putative Vn-binding surface proteins in those particular strains. Taken together, our data demonstrate that the differences in KatA-dependent variations of Vn binding are not due to changes in the amino acid sequence of KatA but a result of varying surface exposure in addition to other putative Vn-binding proteins in some strains.

### KatA confers increased resistance to complement-mediated attack

Many pathogens capitalise on components of the host’s ECM to enhance adherence or evade the immune response. Vn binds to integrins via its RGD motif and can therefore mediate adherence to host tissues^8^. We tested adherence of *H. pylori* KR697 and CCUG18943 and their isogenic *katA* mutants to the gastric adenocarcinoma cell line AGS in the presence or absence of Vn (Supplementary Fig. S3). However, there was no significant difference between adherence of wild type and mutant strains irrespective of the addition of Vn, suggesting that the KatA-Vn interaction is irrelevant for adhesion of *H. pylori* to the gastric epithelium. In fact, the *H. pylori* adhesin CagL binds integrins via an RGD motif 33, which makes the use of Vn as bridging molecule dispensable.

**Figure 7:**
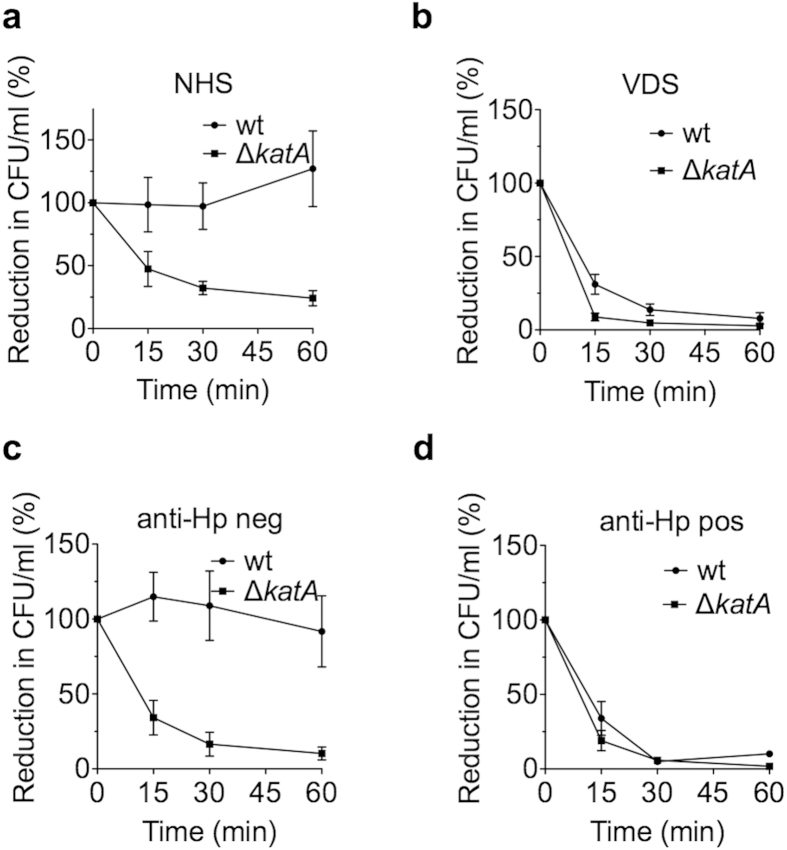
KatA increases complement resistance in a vitronectin-dependent manner. The resistance to serum complement of KR697 wt and Δ*katA* was tested in a series of assays using 5% normal human serum (NHS) of unknown anti-*H. pylori* antibody status (**a**), NHS of unknown anti-*H. pylori* IgG status, which was depleted from vitronectin (VDS) (**b**), NHS of donors negative for anti-*H. pylori* IgG (**c**), and NHS of donors positive for anti-*H. pylori* IgG (**d**). Survival rates at 15, 30 and 60 min after addition of serum were determined by counting colony forming units (CFU). Depicted is the reduction in CFU/ml in percent. Results are the mean and SE of at least three independent experiments performed in technical duplicate.

Another possible outcome of Vn binding is increased serum resistance, due to the inhibitory effect of Vn on the terminal pathway of the complement system^8^. Even though *H. pylori* does typically not enter the bloodstream, it is exposed to complement, since both complement factors and regulators, including Vn, are present in the gastric epithelium during *H. pylori* infection^34^,^35^. Furthermore, activation of complement in the presence of *H. pylori* has been demonstrated *in vivo* and *in vitro*^31^,^36^. We obtained normal human serum (NHS) from healthy volunteers and performed serum killing assays. The survival rates of *H. pylori* KR697 and the KatA-deficient KR697Δ*katA* in 5% NHS were determined over a time course of 60 min (Fig. 7a). Strikingly, no killing was observed in the wild type, but rather a slight increase in colony forming units (CFU) towards the end of the assay. On the contrary, *H. pylori* Δ*katA* showed a marked decrease of 53% already after 15 min, which was, however, not statistically significant. At 30 min CFU counts were significantly reduced to 32% when compared to the wt (*p* < 0.05). After 60 min, the CFU/ml was reduced to 24% of the starting value (*p* < 0.001). Heat inactivated control serum, did not kill any of the strains (data not shown).

To corroborate that the observed difference of *H. pylori* wild type and Δ*katA* with respect to complement resistance is due to the ability of KatA to interact with Vn, we depleted serum of the same pool from Vn and performed another killing assay (Fig. 7b). Indeed, removal of Vn resulted in killing of the wild type confirming that KatA confers increased complement resistance in a Vn-dependent manner (Fig. 7b).

To elucidate whether previous exposure to *H. pylori*, *i.e.*, the presence of anti-Hp Abs, would influence the outcome of the KatA-Vn mediated complement resistance, we obtained sera from a different pool of donors, and tested them for the presence of anti-*H. pylori* IgG using a commercial whole cell ELISA and pooled the sera according to their positive or negative status. We thereafter investigated the survival of *H. pylori* KR697 wt and Δ*katA* in 5% NHS for 60 min (Fig. 7c,d). When using the anti-*H. pylori* IgG negative serum, no killing of *H. pylori* KR697 wt was observed but bacterial counts were significantly reduced for *H. pylori* Δ*katA* (Fig. 7c) at all time points (*p* < 0.001). We obtained similar results for strain CCUG18943 (Supplementary Fig. S4), even though both the wt and mutant were generally more sensitive to serum. Nevertheless, there were statistically significant differences in survival at all time points (*p* < 0.001). Intriguingly, there was no significant difference in serum sensitivity between KR497 wt and its corresponding Δ*katA* mutant, and we observed moderate survival of both strains (Supplementary Fig. S4). We hypothesised that in this particular “low” Vn-binding strain (Fig. 2, Supplementary Fig. S1), Vn binding was not the predominant strategy to avoid complement attack. In fact, there are other complement regulators such as Factor H or C4b binding protein (C4BP), that, like Vn, are regularly attracted by bacteria to circumvent complement-mediated killing^37^. If such an additional mechanism was in place for KR497 it might easily mask the effect of the KatA-dependent Vn binding.

Next, we wanted to test survival of bacteria in anti-Hp positive serum. Intriguingly, in the presence of specific IgGs directed against *H. pylori,* we observed killing of both the wild type and Δ*katA* strain with no statistically significant differences in the survival rates at any given time point (Fig. 7d). This observation was in agreement with other studies^38^,^39^, which observed an overall higher survival rate of *H. pylori* in serum devoid of detectable anti-*H. pylori* IgG than in serum, which contained specific antibodies. A possible explanation is the different modes of activation of the complement system. The alternative pathway, which is independent of specific antibodies, is constitutively activated at a low level. Presence of specific IgG or IgM, however, results in strong activation of the complement cascade via the classical pathway^11^. Thus, we conclude, that the KatA-Vn dependent complement resistance is highly relevant when complement is activated via the alternative pathway, but not sufficient to counteract the stronger activation through the classical pathway that might occur in patients that previously have been exposed to *H. pylori*.

**Figure 8:**
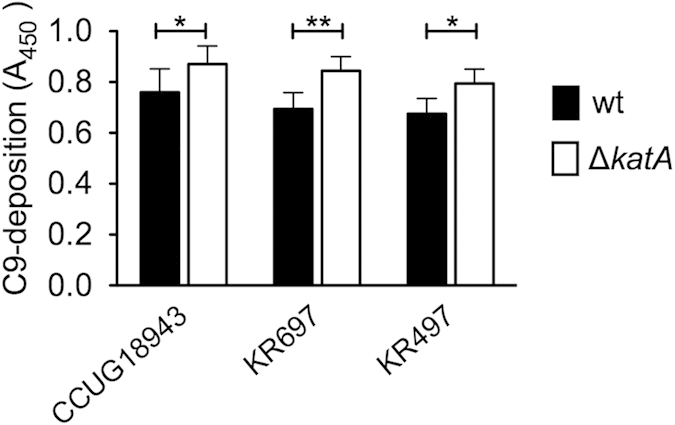
Deposition of MAC is increased in *H. pylori* strains lacking *katA*. Deposition of C9 on CCUG18943, KR697, and KR497 wt and Δ*katA* strains was analysed by ELISA. Bacteria were coated onto 96-well Maxisorp plates and incubated with 5% NHS negative for anti-Hp IgG. C9 deposition was detected using specific antibodies. Statistically significant differences were determined using one-way ANOVA and Bonferroni’s post-test where (*) equals *p* < 0.05 and (**) equals *p* < 0.01. Data presented are the mean and SE of three independent biological replicates performed in duplicates.

As mentioned above, Vn interferes with formation of the MAC. Consequently, if *H. pylori* used KatA to bind Vn, MAC deposition should be decreased on *H. pylori* wt strains compared to those in which *katA* has been deleted. We therefore conducted an ELISA, in which we determined C9 deposition on wt and Δ*katA* of CCUG18943, KR697, and KR497 after incubation with 5% anti-Hp IgG negative NHS (Fig. 8). Indeed, we observed a statistically significant increased MAC deposition on all three Δ*katA* strains further confirming a role of KatA in complement resistance. Surprisingly, this finding also included strain KR497, where no significant difference was seen between wt and Δ*katA* in the complement killing assays. This discrepancy is best explained with the different nature of the two assays. While the complement killing assay, which is based upon CFU count, gives information on differences in the number of culturable bacteria, the ELISA does not discriminate between viable but not culturable (VBNC) and culturable forms. It is currently impossible to revive coccoid VBNC *H. pylori* under laboratory conditions, but there are hints that this form is still relevant for pathogenesis in the host^40^. Hence we cannot exclude a possible effect of a KatA-dependent complement resistance related to Vn for strain KR497, even though it might not be visible in a CFU based assay. Taken together, our data on complement resistance clearly demonstrate the importance of KatA in high and medium Vn-binding strains and even indicate involvement of KatA in Vn-mediated complement resistance in low Vn-binding strains.

This is the first report on identification of a *H. pylori* Vn-binding protein and the beneficial consequences for the bacterium resulting from this interaction. Moreover, we have identified an alternative function of *H. pylori* catalase with implications for evasion of the innate immune response and consequently virulence.

## Methods

### Bacterial strains and culture conditions

Bacterial strains used are listed in supplementary Table S2. *H. pylori* was grown microaerophilic at 37 °C on GC agar or in Brucella broth supplemented with 1% haemoglobin, 10% horse blood, and 1% Vitox (OXOID). *E. coli* BL21(DE3) was grown at 37 °C in LB or on LB-agar. Liquid cultures were grown at 200 rpm. Where applicable, 50 μg/ml kanamycin or 1 mM isopropyl β-D-1-thiogalactopyranoside (IPTG) was added.

### Two-dimensional gel electrophoresis and far-Western blotting

Bacteria outer membrane proteins (OMPs) were prepared as described in Alteri and Mobley^41^. Two-dimensional (2D)-gel electrophoretic separation of OMPs was performed essentially as described before^42^. OMPs (1mg) were incubated with 50 U benzonase at 25 °C for 30 min to digest nucleic acid. Then, samples were solubilized for 1 h in 400 μl of freshly prepared rehydration buffer (5 M urea, 2 M thiourea, 2% (w/v) CHAPS, 65 mM dithiothreitol (DTT), 0.5% (v/v) Pharmalyte pH 3–10, 1 mM phenylmethanesulfonylfluoride (PMSF) and 4 mM -(2-aminoethyl) benzenesulfonyl fluoride hydrochloride). Samples were centrifuged at 8,000 ×*g* for 10 min to remove any precipitates. First dimension separation by isoelectric focusing (IEF) was performed using the following program: 30 V for 0.30 h (15Vhr), 500 V for 0.30 h (250 Vhr), 1,000 V for 0.30 h (500 Vhr), 2,600 V for 0.15 h (650 Vhr), 3,500 V (875 Vhr) and 5,000 V for 1.00 h (5,000 Vhr) resulting in a total voltage of 7291 Vhr, with maximum current of 50 μA per strip. Focused strips were equilibrated in equilibration buffer (50 mM Tris-HCl pH8.8, 6 M urea, 30% glycerol, 2% (w/v) SDS, 20 mM DTT, 0.002% bromophenol blue) for 15 min followed by incubation in the same buffer but replacing DTT with 20 mM iodoacetamide. For second dimension separation, strips were placed on 12% (v/v) SDS-polyacrylamide and proteins separated at 70 V for 15 min and 150 V for 40 min. Protein spots were visualized by Coomassie blue staining. Targeted Coomassie stained protein spots were manually excised and identified by Matrix-assisted laser desorption/ionisation-time of flight mass spectrometry (MALDI-TOF-MS) (Protein Analysis Service, Alphalyse). For far-Western blotting immunoassays, proteins were transferred to a polyvinylidenedifluoride membrane. Membranes were blocked in PBS containing 1.5% ovalbumin and probed with 3 μg/ml human Vn. Bound Vn was detected with sheep anti-Vn IgG (BioRad) and HRP-conjugated donkey anti-sheep/goat IgG (AbD Serotec).

### Construction of *katA* deletion mutants

All primers and plasmids used in this study are listed in supplementary Table S3. Deletion mutants of *katA* were obtained for *H. pylori* strains KR697, KR497 and CCUG18943 by homologous recombination. Constructs containing a kanamycin cassette and 1000 bp flanks were created by overlap extension PCR. All primers used are listed in supplementary Table S2. The kanamycin cassette was amplified from pET26 using primers Kan_F and Kan_R. Downstream flanks of *katA* were amplified from genomic DNA of the respective strain using primers DF_*katA*_F2_Kan and DF_*katA*_R2. Upstream flanks of *katA* were amplified using primer pair UF_*katA*_F1 and UF_*katA*_R6_Kan for KR697 and KR497, and primer pair UF_*katA*_F1 and UF_*katA*_R4 for CCUG18943. For the overlap reaction, 10 ng of each PCR product were used with primer pair UF_*katA*_F1 and DF_*katA*_R2. Overlap PCR products were transformed into the respective *H. pylori* strain using natural competence. Briefly, freshly grown bacteria were resuspended in PBS to an OD_600_ = 5, spotted on GC agar without selection and incubated for 8 h before adding the 0.2 μg of PCR product. Plates were further incubated for 16 h. Growth spots were scraped of the plates, resuspended in PBS, plated on GC plates with kanamycin and incubated for 72 h. Resulting mutants were confirmed by PCR, Western blotting and loss of catalase function.

### Flow cytometry analysis

Clinical isolates of *H. pylori* were grown under microaerophilic conditions in liquid cultures at 37 °C, 200 rpm until mid-log phase. To assess Vn binding, bacteria were incubated with 3 μg of human serum Vn per 10^6^ cells for 1 h at 37 °C in PBS 1% BSA to allow Vn to bind bacteria. To remove unbound Vn, bacteria were washed twice in PBS. The amount of bound Vn was measured by flow cytometry (BD Verse) using a sheep anti-human Vn Ab (BioRad) and a fluorescein isothiocyanate (FITC)-conjugated anti-sheep pAb (BioRad) as a secondary layer. To determine KatA surface exposure, bacteria were blocked with PBS 1% BSA and incubated with 1 μg rabbit-anti-KatA for 1 h on ice. Bound anti-KatA was detected by flow cytometry using FITC-conjugated swine anti-rabbit pAb (Dako). Controls without Vn or Abs were included in all experiments to exclude non-specific binding. Data analysis was performed on FACS DIVA.

### DNA manipulations and cloning

All primers and plasmids are listed in supplementary Table S3. Full length *katA* and *katA*-fragments were amplified by PCR from CCUG18943 genomic DNA using the following primer combinations: katA_for2 and katA_rev505 or katA_rev49 for full length *katA* or *katA*^1–49^, respectively; katA_for51 and katA_rv505 or katA_rev488 for *katA*^*51–505*^ or *katA*^*51–488*^, respectively. Finally, combinations katA_for350 and katA_rev505, and katA_for401 and katA_rev488 were used for *katA*^*350–505*^ and *katA*^*401–488*^. Resulting products were digested with NdeI and XhoI and ligated into pET26 previously digested with the same enzymes. All constructs were confirmed by sequencing (MWG Eurofins). Constructs containing Vn-fragments were obtained as described earlier^43^.

### Purification of proteins and protein fragments

Activated Vn was purified from serum of healthy human volunteers as described in^44^. Briefly, serum was depleted of other heparin binding components before the heparin binding ability of Vn was activated by treatment with urease. Vn was thereafter purified by affinity chromatography using heparin as matrix. Recombinant full length Vn and Vn fragments were prepared as described previously^43^. Briefly, HEK293T cells were grown in three triple flasks (Nunc) to 80% confluence using advanced DMEM supplemented with penicillin (100 U/μl) and streptomycin (100 μg/ml) and 1% FCS at 37 °C with 5% CO_2_. Transfected cells were incubated for 3 d at 37 °C with 5% CO_2_ followed by harvesting of supernatants. A similar volume of advanced DMEM was once again added to the cells and the procedure was repeated after 3 d. His-tagged Vn was secreted into the medium and purified by Ni-NTA chromatography. Recombinant KatA or KatA-fragments were expressed in BL21(DE3). Liquid cultures were grown to an OD_600_ = 0.5, induced with 1 mM IPTG, and grown for further 3 to 4 h before harvest by centrifugation. Cell pellets were resuspended in binding buffer (20 mM NaPO_4_; 500 mM NaCl; 20 mM imidazole; pH 7.4), lysed by sonication, and centrifuged for 20 min at 4 °C and 23200 x *g* to separate soluble and insoluble fraction. Proteins were purified by Ni-affinity chromatography on His-Trap FF columns (GE Healthcare) and eluted in binding buffer supplemented with 500 mM imidazole. Fragment KatA^350–505^ was further purified by size exclusion using a Superdex 75 (10/300) column (GE Healthcare). Eluted fractions were analysed by SDS-PAGE and Coomassie staining. Fractions containing the desired protein were dialysed against 50 mM NaPO_4_, 150 mM NaCl, pH 7.4 and stored at 4 °C until further use. For KatA1-49 the pellet containing the insoluble fraction was dissolved in binding buffer with 8 M urea and proteins were then refolded by dialysis against binding buffer prior to purification. All other proteins were purified from the soluble fraction. Catalase activity of full length KatA as well as KatA fragments containing the active site (KatA^51–505^ and KatA^51–488^) was confirmed visually using H_2_O_2_. Anti-KatA IgG was purified from serum from rabbits immunized with recombinant KatA using KatA-coupled CNBr-activated Sepharose (GE healthcare). Bound anti-KatA polyclonal antibodies (pAb) were eluted with 100 mM glycine, pH 3.0 and immediately neutralized with 1.5 M Tris-HCl, pH 9.0. To reduce unspecific background anti-KatA IgG were absorbed with CCUG18943Δ*katA* prior to use.

### Assessment of protein interaction by enzyme-linked immunosorbent assay (ELISA)

Polysorb 96-well plates (Nunc) were coated with 100 μl of 100 nM full length KatA or KatA fragments dissolved in coating buffer (100 mM Tris-HCl; 150 mM NaCl; pH 9.0) overnight at 4 °C. All following incubation steps were carried out at room temperature for 1 h. Plates were washed 4 times with PBS + 0.05% Tween20 between all incubations. First, plates were blocked with 2.5% BSA in PBS and thereafter incubated with recombinant Vn or Vn fragments at concentrations between 0–80 nM. Binding was detected using sheep anti-human-Vn (BioRad) pAb followed by horseradish-peroxidase (HRP)-conjugated rabbit anti-sheep pAb (AbD Serotec). To assess binding of KatA to monomeric or polymeric Vn, 50 nM Vn was coated to Polysorb plates as described above. After blocking, plates were incubated with 0–80 nM KatA. Bound KatA was detected using rabbit-anti-KatA pAb followed by HRP-conjugated swine anti-rabbit IgG (Dako). Data presented are means of at least three independent experiments performed in technical triplicates. Assays without Vn or KatA and controls consisting of secondary Abs only were included in all experiments.

### BioLayer Interferometry

BioLayer Interferometry was carried out on an Octed RED96 platform (ForteBio). Vn^80–396^ was immobilised onto AR2G sensors at pH 5 using the Amine Coupling Reagent kit (ForteBio) according to the manufacturer’s instructions. Binding was tested using a 1 in 3 dilution series of KatA (50–0.055 nM) in PBS, 0.05% Tween20 (Sigma). A buffer only control served as reference well. Data analysis was performed using ForteBio Data Analysis 8.1. and Prism GraphPad version 5. Steady state analysis was performed based on the equilibrium response.

### Serum resistance assays

Normal human serum (NHS) was obtained from healthy volunteers of unknown *H. pylori* infection status. Depletion of Vn was accomplished by running serum against anti-Vn-coupled CNBr Sepharose as described previously^45^. Western blotting was used to confirm depletion of Vn and to ensure that levels of serum-antibodies were unaffected by the depletion process. Sera were tested for presence of IgG against *H. pylori* using the whole cell anti-*H. pylori* (ATCC43504) ELISA (Euroimmun) and pooled according to the IgG status. Heat-inactivation was performed for 30 min at 56 °C. To investigate complement-mediated killing, *H. pylori* KR697 or KR697Δ*katA* grown in liquid culture were incubated in DGVB++ buffer (72 mM NaCl, 0.9 mM sodium barbital, 1.55 mM barbituric acid, 0.1% gelatine, 1 mM MgCl_2_, 0.15 mM CaCl_2_, 2.5% dextrose) with 5% serum at 37  °C, 5% CO_2_ for 60 min. To exclude effects of the incubation conditions on the viability of the bacteria, serum-free controls were run in parallel and CFUs determined at the start and end of the assay (data not shown). Samples were plated 0, 15, 30, and 60 min after serum was added and the colony forming units (CFU/ml) were determined.

### Measurement of C9 deposition by ELISA

Bacteria were grown to mid-log phase, harvested, washed, and diluted to 2*10^7^ CFU/ml in PBS. 1*10^7^ CFU in 50 μl bacterial suspension were immobilised over night at 4 °C in MaxiSorp 96 well plates (NUNC). Next day, plates were washed thrice using immunowash (50 mM Tris, 0.1% Tween 20; 150 mM NaCl) and blocked with quench (3% fish gelatine and 0.02% NaN_3_ in immunowash) for 1 h at 37 °C. After every incubation step, the plates were washed three times with 250 μl/well immunowash. Five per cent NHS in DGVB++ was added and plates incubated for 1 h at 37 °C. Deposited C9 was detected using goat anti-human C9 (CompTech) followed by rabbit anti-goat horse-raddish conjugated IgG. The ELISA was developed by using 50 μl of 3′,3′,5′,5′-Tetramethylbenzidine (TMB) Liquid Substrate System for ELISA (Sigma) and stopped by adding 50 μl 0.5 M H_2_SO_4_. Data presented are the means and SE of three independent biological replicates, each performed in technical duplicate.

### Statistical analysis

All statistical analyses were performed using Graph-Pad Prism^®^ version 5.0 (GraphPad Software, La Jolla, CA). To determine statistical differences between obtained data sets, Student’s *t*-test (flow cytometry analysis of Vn-binding of wt versus Δ*katA* strains), one-way ANOVA with Bonferroni’s post-test (ELISA), or two-way ANOVA with Bonferroni’s post-test (serum resistance assays) was performed. Differences between experiments were considered statistically significant for *p* < 0.05.

## Additional Information

**How to cite this article**: Richter, C. *et al.* Moonlighting of *Helicobacter pylori* catalase protects against complement-mediated killing by utilising the host molecule vitronectin. *Sci. Rep.* **6**, 24391; doi: 10.1038/srep24391 (2016).

## Supplementary Material

Supplementary Information
