# Supplementary material for: Moonlighting of Helicobacter pylori catalase protects against complement-mediated killing by utilising the host molecule vitronectin
Source: Sci Rep. 2016 Apr 18;6:24391. doi: 10.1038/srep24391 (PMC4834553; doi:10.1038/srep24391)
Supplement: Supplementary Information [file srep24391-s1.pdf]

Supplementary Information

Moonlighting of *Helicobacter pylori* catalase protects against complement-mediated killing by utilizing the host molecule vitronectin

Corinna Richter[1], Oindrilla Mukherjee[1], David Ermert[2], Birendra Singh[1], Yu-Ching Su[1], Vaibhav Agarwal[2], Anna M. Blom[2] and Kristian Riesbeck[1*]

[1] Clinical Microbiology and [2] Medical Protein Chemistry, Department of Translational Medicine, Lund University, SE-205 02 Malmö, Sweden

Corresponding author: Dr. Kristian Riesbeck, Clinical Microbiology, Department of Translational Medicine, Lund University, Jan Waldenströms gata 59, SE-205 02 Malmö, Sweden. Phone: +46-40-336234. E-mail: kristian.riesbeck@med.lu.se

Supplementary Table 1: Correlation of Vn binding and KatA surface exposure

	Vn binding (%)		anti-KatA binding (%)	
**Strain**	**Mean**	**SD**	**Mean**	**SD**
CCUG18943	91.42	4.89	58.36	1.48
KR557	88.13	4.38	27.56	15.19
CCUG17875	83.73	3.35	56.19	13.94
KR560	75.06	5.60	59.96	6.59
KR697	69.47	3.07	47.34	26.76
KR524	55.20	10.59	21.75	2.81
CCUG19106	50.60	14.84	nd	nd
KR698	38.38	13	6.47	3.88
KR496	33.31	84	32.38	16.56
KR558	30.60	1.91	nd	nd
KR549	23.47	3.17	23.93	4.71
KR548	18.63	4.27	21.07	3.57
KR497	15.20	3.48	5.10	0.51
CCUG18943ΔkatA	na	na	28.6	6.39
KR697ΔkatA	na	na	9.31	4.12
KR497ΔkatA	na	na	4.96	2.83

Supplementary Table 2: Bacterial strains

**Strain**	**Description**	**Reference/ Source**
E. coli		
DH5α	F– Φ80*lac*ZΔM15 Δ(*lac*ZYA-*arg*F) U169 *rec*A1 *end*A1 *hsd*R17 (rK–, mK+) *pho*A *sup*E44 λ– *thi*-1 *gyr*A96 *rel*A1	Invitrogen
BL21 (DE3)	*fhuA2 [lon] ompT gal (λ DE3) [dcm]ΔhsdSλ DE3 = λ sBamHI oΔEcoRI-B int::(lacI::PlacUV5::T7 gene1) i21 Δnin5*	Novagen
H. pylori		
CCUG18943	$cagA^+$	Culture Collection, University of Göteborg, Sweden (CCUG)
CCUG18943Δ*katA*	*katA* deficient CCUG18943	This study
KR697	clinical isolate, $cagA^+$	This study
KR697Δ*katA*	*katA* deficient KR697	This study
KR497	clinical isolate, $cagA^+$	This study
KR497Δ*katA*	*katA* deficient KR497	This study
KR496	clinical isolate, $cagA^+$	This study
KR524	clinical isolate, $cagA^+$	This study
KR548	clinical isolate, $cagA^+$	This study
KR549	clinical isolate, $cagA^+$	This study
KR557	clinical isolate, $cagA^+$	This study
KR558	clinical isolate, $cagA^+$	This study
KR560	clinical isolate, $cagA^+$	This study
KR698	clinical isolate, $cagA^+$	This study
CCUG17875	$cagA^+$	CCUG
CCUG19106	$cagA^-$	CCUG

Supplementary Table 3: Primers and plasmids

**Plasmids**	
pET26	Protein expression vector, Km^R
pET26*katA*	Expression vector for *H. pylori* CCUG18943 KatA
pET26*katA*1-49	Expression vector for *H. pylori* CCUG18943 KatA aa 1-49 fragment
pET26*katA*51-505	Expression vector for *H. pylori* CCUG18943 KatA aa 51-505 fragment
pET26*katA*51-488	Expression vector for *H. pylori* CCUG18943 KatA aa 51-488 fragment
pET26*katA*51-400	Expression vector for *H. pylori* CCUG18943 KatA aa 51-400 fragment
pET26*katA*350-505	Expression vector for *H. pylori* CCUG18943 KatA aa 350-505 fragment

**Oligonucleotides** (5' – 3')	
*katA*_for2	ATGCCTGACATATGGTTAATAAAGATGTGAAACAAACCACTGC
*katA*_for51	ATATCTGACATATGCCTGAAAGGGTAGTGCATGCTAAAGGAAGC
*katA*_for350	ATGCCTGACATATGAATTATCCTCAAATACCGGTTAATAAACCAAGA
*katA*_for401	ATGCCTGACATATGAAGTTCAACTTAGCTCATATTGAGAAAGAG
*katA*_rev49	ATCCTGACTCGAGCCTTTCTCTGTCAAACGCTGCTAACTT
*katA*_rev488	ATTCTGACTCGAGCTTTTGGTGTTTTTCAAGAGCTTTTTTAACTCCCTC
*katA*_rev505	ATATGACTCGAGCTTTTTCTTTTTTGTGTGGTGCATGTCTTTTCC
Kan_F	ATGAGCCATATTCAACGGGAAAC
Kan_R	TTAGAAAAACTCATCGAGCATCAA
UF_*katA*_F1	GCGTTATCTCGTGTCATGTTCCAGC
UF_*katA*_R6_Kan	TCCCGTTGAATATGGCTCATCTTCTTTCCTTTATTTGATTTAAATTTT
UF_*katA*_R4_Kan	TCCCGTTGAATATGGCTCATCTTTTTTCCTTTATTTGACTTCAAATCT
DF_*katA*_F2_Kan	TGCTCGATGAGTTTTTCTAACCCTTTTCTTTAAGCGTTCTTATTTTT
DF_*katA*_R2	ATTCCTAGATTTCATGCCTTTAGAC

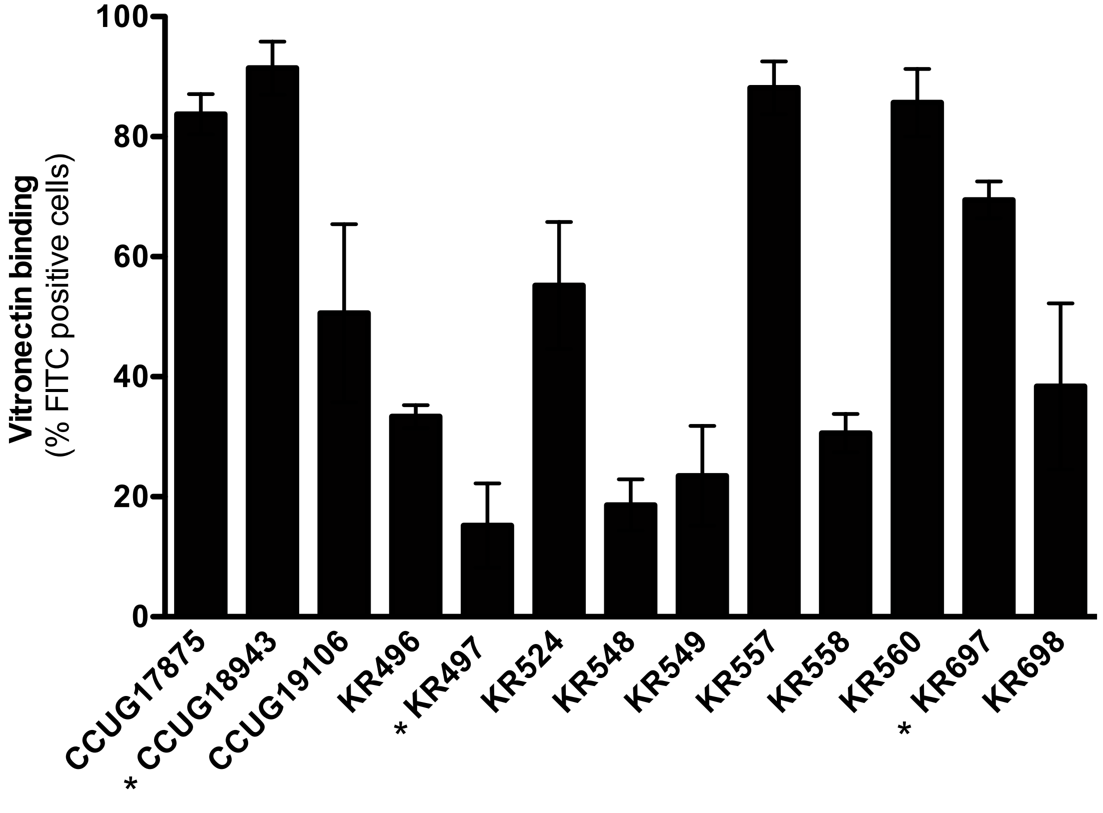

Supplementary Figure S1. Vitronectin binding differs between *H. pylori* isolates. Clinical isolates of *H. pylori* were incubated with 3 μg of vitronectin purified from human serum. The percentage of vitronectin-binding was determined by flow-cytometry using FITC-labeled α-Vn antibody. Data presented are the mean and SD of at least three independent experiments. Asterisks indicate the three strains chosen for further analyses.

```
18943        MVNKDVKQTTAFGAPVWDDNNVITAGPRGPVLLQSTWFLEKLAAFDRERIPERVVHAKGS 60
697          MVNKDVKQTTAFGAPVWDDNNVITAGPRGPVLLQSTWFLEKLAAFDRERIPERVVHAKGS 60
26695        MVNKDVKQTTAFGAPVWDDNNVITAGPRGPVLLQSTWFLEKLAAFDRERIPERVVHAKGS 60
497          MVNKDVKQTTAFGAPVWDDNNVITAGPRGPVLLQSTWFLEKLAAFDRERIPERVVHAKGS 60

18943        GAYGTFTVTKDITKYTKAKIFSKVGKKTECFFRFSTVAGERGSADAVRDPRGFAMKYYTE 120
697          GAYGTFTVTKDITKYTKAKIFSKVGKKTECFFRFSTVAGERGSADAVRDPRGFAMKYYTE 120
26695        GAYGTFTVTKDITKYTKAKIFSKVGKKTECFFRFSTVAGERGSADAVRDPRGFAMKYYTE 120
497          GAYGTFTVTKDITKYTKAKIFSKVGKKTECFFRFSTVAGERGSADAVRDPRGFAMKYYTE 120

8943         EGNWDLVGNNTPVFFIRDAIKFPDFIHTQKRDPQTNLPNHDMVWDFWSNVPESLYQVTWV 180
697          EGNWDLVGNNTPVFFIRDAIKFPDFIHTQKRDPQTNLPNHDMVWDFWSNVPESLYQVTWV 180
26695        EGNWDLVGNNTPVFFIRDAIKFPDFIHTQKRDPQTNLPNHDMVWDFWSNVPESLYQVTWV 180
497          EGNWDLVGNNTPVFFIRDAIKFPDFIHTQKRDPQTNLPNHDMVWDFWSNVPESLYQVTWV 180

18943        MSDRGIPKSFRHMDGFGSHTFSLINAKGERFWVKFHFETMQGVKHLTNEEAAEIRKYDPD 240
697          MSDRGIPKSFRHMDGFGSHTFSLINAKGERFWVKFHFETMQGVKHLTNEEAAEVRKYDPD 240
26695        MSDRGIPKSFRHMDGFGSHTFSLINAKGERFWVKFHFHTMQGVKHLTNEEAAEVRKYDPD 240
497          MSDRGIPKSFRHMDGFGSHTFSLINAKGERFWVKFHFHTMQGVKHLTNEEAAEIRKHDPD 240

18943        SNQRDLFNAIARGDFPKWKLSIQVMPEEDAKKYRFHPFDVTKIWYLQDYPLMEVGIVELN 300
697          SNQRDLFNAIARGDFPKWKLSIQVMPEEDAKKYRFHPFDVTKIWYLQDYPLMEVGIVELN 300
26695        SNQRDLFNAIARGDFPKWKLSIQVMPEEDAKKYRFHPFDVTKIWYLQDYPLMEVGIVELN 300
497          SNQRDLFNAIARGDFPKWKLSIQVMPEEDAKKYRFHPFDVTKIWYLQDYPLMEVGIVELN 300

18943        KNPENYFAEVEQAAFSPANVVPGIGYSPDRMLQGRLFSYGDTHRYRLGVNYPQIPVNKPR 360
697          KNPENYFAEVEQAAFSPANVVPGIGYSPDRMLQGRLFSYGDTHRYRLGVNYPQIPVNKPR 360
26695        KNPENYFAEVEQAAFSPANVVPGIGYSPDRMLQGRLFSYGDTHRYRLGVNYPQIPVNKPR 360
497          KNPENYFAEVEQAAFSPANVVPGIGYSPDRMLQGRLFSYGDTHRYRLGVNYPQIPVNKPR 360

18943        CPFHSSSRDGYMQNGYYGSLQNYTPSSLPGYKEDKSARDPKFNLAHIEKEFEVWNWDYRA 420
697          CPFHSSSRDGYMQNGYYGSLQNYTPSSLPGYKEDKSARDPKFNLAHIEKEFEVWNWDYRA 420
26695        CPFHSSSRDGYMQNGYYGSLQNYTPSSLPGYKEDKSARDPKFNLAHIEKEFEVWNWDYRA 420
497          CPFHSSSRDGYMQNGYYGSLQNYTPSSLPGYKEDKSARDPKFNLAHIEKEFEVWNWDYRA 420

18943        EDSDYYTQPGDYYRSLPADEKERLHDTIGESLAHVTHKEIVDKQLEHFKKADPKYAEGVK 480
KR697        EDSDYYTQPGDYYRSLPADEKERLHDTIGESLAHVTHKEIVDKQLEHFKKADPKYAEGVK 480
26695        DDSDYYTQPGDYYRSLPADEKERLHDTIGESLAHVTHKEIVDKQLEHFKKADPKYAEGVK 480
497          DDSDYYTQPGDYYRSLPADEKERLHDTIGESLAHVTHKEIVDKQLEHFKKADPKYAEGVK 480

18943        KALEKHQKMMKDMHGKDMHHTKKKK 505
697          KALEKHQKMMKDMHGKDMHHTKKKK 505
26695        KALEKHQKMMKDMHGKDMHHTKKKK 505
497          KALEKHQKMMKDMHGKDMHHTKKKK 505
```

Supplementary Figure S2. KatA sequences of strong, intermediate and weak vitronectin binding *H.* pylori strains show only small differences. KatA amino acid sequences of *H. pylori* CCUG18943, KR697, Hp22965, and KR497 were aligned using Clustal Omega (http://www.ebi.ac.uk/Tools/msa/clustalo/). The putative vitronectin-binding site is marked in grey and positions where changes in the amino acid sequence occur are marked in yellow.

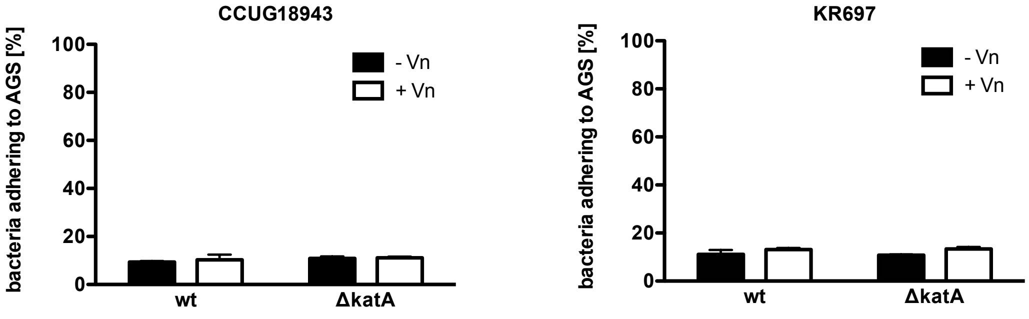

Supplementary Figure S3. The presence of KatA and vitronectin does not affect adhesion of *H. pylori* to AGS cells. Gastric adenocarcinoma cells (AGS) were incubated with *H. pylori* at multiplicity of infection (MOI) 100 in the presence or absence of vitronectin. Cells and bacteria were incubated for a period of 3 h, cells were lysed and dilutions of bacteria plated on GC plates for CFU counts. The relative number of bacteria, which attached to the AGS cells is shown. Data presented are the mean and SD of three independent experiments performed in technical triplicate.

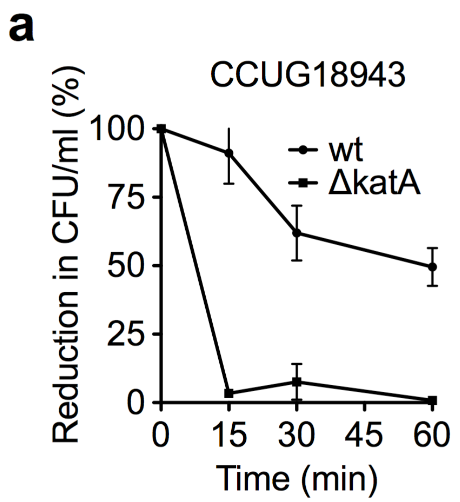

Supplementary Figure S4: KatA confers increased complement resistance in high-Vn binding strains. The resistance of CCUG18943 (a) and KR497 (b) wt and D*katA* to serum complement was tested in a series of assays using 5% normal human serum (NHS) of donors negative for anti-*H. pylori* IgG. Survival rates at 15, 30 and 60 min after addition of serum were determined by plate count. Depicted is the reduction in CFU/ml in percent. Results are the mean and SE of at least three independent experiments performed in technical duplicate.

Supplementary Methods

H. pylori *adhesion to AGS cells*
The human gastric adenocarcinoma cell line (AGS) was maintained in F-12 medium (GIBCO), 10% FBS at 37°C, 5% CO_2. Cells were routinely subcultured every 2–3 days. AGS cells were seeded into wells of cell culture dishes and grown till confluency and starved overnight prior to infection. Bacteria were grown on GC plates for 72 hours. To observe the effect of vitronectin on adherence of *H. pylori* to AGS cells, serum purified Vitronectin was added at a concentration of 10 μg/ml at 4°C for 1 h followed by two washes prior to the addition of *H. pylori* at an MOI of 1:100. Cells were incubated for a period of 3 hours after infection, washed thoroughly and cells were collected by treatment with citric acid buffer. Cells were then lysed with glass beads, vortexed, and serial dilutions plated on GC agar plates. Colony counts were performed after 72 h incubation.
